# Impact of Paxlovid on in-hospital outcomes and post-COVID-19 condition in adult patients infected with SARS-CoV-2 Omicron variant: A non-randomized controlled clinical trial

**DOI:** 10.1097/MD.0000000000036714

**Published:** 2023-12-22

**Authors:** Jianchao Xu, Jinzhong Song, Ziyu Xie, Jie Yang, Di Wu, Fengshuang Liu, Yinuo Zhao, Hongmin Zang, Yubin Zhao

**Affiliations:** a Hebei University of Chinese Medicine, Shijiazhuang, China; b Shijiazhuang People’s Hospital, Shijiazhuang, China; c The Traditional Chinese Medicine Hospital of Shijiazhuang, Shijiazhuang, China; d Hebei Medical University, Shijiazhuang, China; e Hebei General Hospital, Shijiazhuang, China; f Hebei Academy of Chinese Medical Sciences, Shijiazhuang, China; g School of Biological Sciences, Faculty of Biology, Medicine and Health, The University of Manchester, Manchester, UK; h North China University of Science and Technology, Tangshan, China.

**Keywords:** adult, clinical trial, COVID-19, Omicron, Paxlovid, SARS-CoV-2

## Abstract

**Background:**

Nirmatrelvir plus ritonavir (Paxlovid) have been used in the treatment of adult patients with mild-to-moderate coronavirus disease 2019 (COVID-19). This study aimed to evaluate the impact of Paxlovid on in-hospital outcomes and post-COVID-19 condition in Chinese adult patients infected with severe acute respiratory syndrome coronavirus 2 (SARS-CoV-2) Omicron variant.

**Methods:**

This non-randomized clinical controlled trial recruited patients infected with SARS-CoV-2 Omicron variant from the designated hospital for treating COVID-19 between November 5 and November 28, 2022, in Shijiazhuang, China. Participants were administered Paxlovid (300 mg of nirmatrelvir and 100 mg of ritonavir orally) or standard treatment. The primary outcome was the nucleic acid shedding time and post-COVID-19 condition.

**Results:**

A total of 320 patients infected with SARS-CoV-2 Omicron variant were included, with mean age of 29.10 ± 7.34 years old. Two hundred patients received Paxlovid. Compared to patients in the standard treatment group, those in Paxlovid group had a significantly shorter nucleic acid shedding time (3.26 ± 1.80 vs 7.75 ± 3.68 days, *P* < .001), shorter days until negative swab test (1.74 ± 1.15 vs 5.33 ± 2.91, *P* < .001), shorter days of first symptoms resolution (4.86 ± 1.62 vs 7.45 ± 2.63, *P* < .001), higher in nucleic acid test negative rate within 3 days [138 (70.77%) vs 14 (11.67%), *P* < .001], higher negative rate within 5 days [174 (89.23%) vs 26 (21.67%), *P* < .001], negative rate within 7 days [185 (94.87%) vs 78 (65.00%), *P* < .001], and were less likely to have post-COVID-19 condition [32 (18.60%) vs 30 (31.57%), *P* = .016]. There was no significant difference in duration of post-COVID-19 condition (43.00 ± 26.00 vs 49.00 ± 26.34 days, *P* = .354) between the 2 groups.

**Conclusion:**

Compared to standard treatment, Paxlovid significantly reduced nucleic acid shedding time, days until negative swab test, and days of first symptoms resolution, as well as improved nucleic acid test negative rate and post-COVID-19 condition.

## 1. Introduction

As an international public health emergency, coronavirus disease 2019 (COVID-19) is one of the greatest threats to human health in the 21st century.^[1,2]^ The disease has caused over 750 million confirmed cases and 6 million deaths worldwide.^[3]^ The Omicron variant is the dominant strain of all the severe acute respiratory syndrome coronavirus 2 (SARS-CoV-2) variants, with twice as many mutations in its spike protein as the Delta variant.^[4–6]^ The Omicron variant is certainly more infectious, yet far less virulent than previous strains, with hospitalization rate of <0.5%.^[7]^ Furthermore, it has faster transmission speed and more significant immune evasion.^[8,9]^

Nirmatrelvir plus ritonavir (Paxlovid) is a new antiviral drug indicated for high risk patients to prevent severe form of COVID-19.^[10]^ Paxlovid is composed of nirmatrelvir, a novel protease inhibitor of the SARS-CoV-2 3C-like protease, and ritonavir, which serves as an inhibitor of cytochrome P450 3A4, reducing the metabolism of nirmatrelvir and increasing its serum level.^[11–13]^ Related studies indicated its good effect on reducing the risk of severe disease and death in patients infected with COVID-19.^[14,15]^ Paxlovid has been approved for emergency use by the United States Food and Drug Administration on December 22, 2021, and conditionally approved for import and use by the China National Medical Products Administration on February 11, 2022. It has been used in epidemic situations in various regions and recommended in the diagnosis and treatment guidelines for COVID-19 in China.^[16,17]^ However, there is currently limited research on the use of Paxlovid in Chinese adults. Furthermore, information on the impact of Paxlovid on the incidence of post-COVID-19 condition after viral nucleic acid turns negative is still scarce.

This study aimed to evaluate impact of Paxlovid on in-hospital outcomes and long-term post-COVID-19 condition in adults patients infected with SARS-CoV-2 Omicron variant.

## 2. Methods

### 2.1. Study design and patients

This non-randomized clinical controlled trial recruited adult patients infected with the Omicron variant of COVID-19 who were admitted to the designated hospital for treating COVID-19 in Shijiazhuang between November 5 and November 28, 2022. The inclusion criteria were: age between 18 and 50 years old; positive nucleic acid result; no previous systemic treatment; mild or moderate COVID-19 within 5 days of onset according to the “Diagnosis and Treatment Guide for COVID-19 (Version 9)”; and voluntary participation and signed written informed consent. Exclusion criteria were: patients with mental illness; participation in other clinical studies; patients with malignant tumors, hemophilia, severe liver or kidney disease, coronary atherosclerotic heart disease, stroke; or patients who were taking medications which were contraindicated for concomitant use with Paxlovid, including alfuzosin, amiodarone, eletriptan.

This study was approved by the Institutional Review Board of the People Hospital of Shijiazhuang (No. 2023008) and conducted in accordance with the ethical principles for medical research involving human subjects established in the Helsinki Declaration, protecting the privacy and confidentiality of personal information of all participants. Written informed consent was obtained from each patient.

### 2.2. Procedure

Demographic variables such as sex, age, height, weight, first symptoms at onset, diagnosis time, and nucleic acid Ct value were collected. All patients received the basic treatment based on the Chinese “Diagnosis and Treatment Guide for COVID-19 (Version 9).” The patients in the Paxlovid group received 300 mg of nirmatrelvir and 100 mg of ritonavir orally, twice a day for 5 consecutive days, while those in the control group received standard treatment for COVID-19.^[16]^ The standard treatment included centralized isolation management, bed rest, close monitoring of vital signs, especially resting and post-activity oxygen saturation. Standardized and effective oxygen therapy measures were given according to patient condition, including nasal catheter, mask oxygen, and nasal high flow oxygen therapy.

After enrolment, patients were required to test nucleic acid daily, and nucleic acid results and clinical symptoms were recorded. The discharge criteria for patients with standard treatment were: at least 3 consecutive days of normal body temperature; obvious improvement in respiratory symptoms and pulmonary imaging; 2 consecutive nucleic acid tests for the N gene and ORF gene Ct values of the SARS-CoV-2 were ≥ 35, or 2 consecutive nucleic acid tests were negative, with a sampling interval of at least 24 hours. Patients in the Paxlovid group were required to collect nucleic acid continuously for at least 7 days and meet the above discharge criteria before being discharged. Three months after discharge, a follow-up survey by telephone questionnaire was conducted to investigate whether the 2 groups of patients had post-COVID-19 condition related to COVID-19 after nucleic acid turned negative upon discharge. Specific symptom manifestations and the duration of symptoms were recorded. In addition, based on the nucleic acid test results, the researchers observed whether patients in the Paxlovid group experienced the rebound results of the nucleic acid test after turning negative during hospitalization. Adverse events during hospitalization, including unforeseeable medical events that occur when patients receive medication, were also recorded. In addition to the observation of the treatment effect in the acute phase, a telephone follow-up study was also conducted 3 months after the patient was discharged from hospital to observe post-COVID-19 condition.

### 2.3. Outcomes

The primary outcome was the nucleic acid shedding time and post-COVID-19 condition. The secondary outcomes were days until negative swab test, days of first symptoms resolution, nucleic acid test negative rate within 3 days, negative rate within 5 days, and negative rate within 7 days.

The nucleic acid shedding time was defined as the number of days from the first positive test to 2 consecutive negative nucleic acid results after enrolment. Days until negative swab test was defined as the time from the first positive COVID-19 nucleic acid test to the first negative throat swab test. Days of first symptoms resolution was defined as the number of days from the exact time of the patient first symptoms to the exact time of the disappearance of symptoms. Post-COVID-19 condition occurred in individuals with a history of probable or confirmed SARS-CoV-2 infection, usually 3 months from the onset, with symptoms that last for at least 2 months and could not be explained by an alternative diagnosis. Common symptoms included, but were not limited to, fatigue, shortness of breath, and cognitive dysfunction, and generally had an impact on daily functioning. Symptoms might be new onset following initial recovery from an acute COVID-19 episode or persist from the initial illness. Symptoms might also fluctuate or relapse over time.^[18]^

### 2.4. Statistical analysis

The statistical analysis was performed using SPSS version 27.0 (IBM, Armonk, NY, USA). Continuous data with a normal distribution were described as means ± standard deviations and analyzed using t-test. Categorical data were described as n (%) and analyzed using the Pearson chi square test or Fisher exact test. Multivariable logistic/Cox proportional hazards regression analysis were performed to determine the impact of Paxlovid on occurrence of post-COVID-19 condition and the duration of post-COVID-19 symptoms. Two-sided *P* values < .05 were considered statistically significant.

## 3. Results

**Figure 1.**
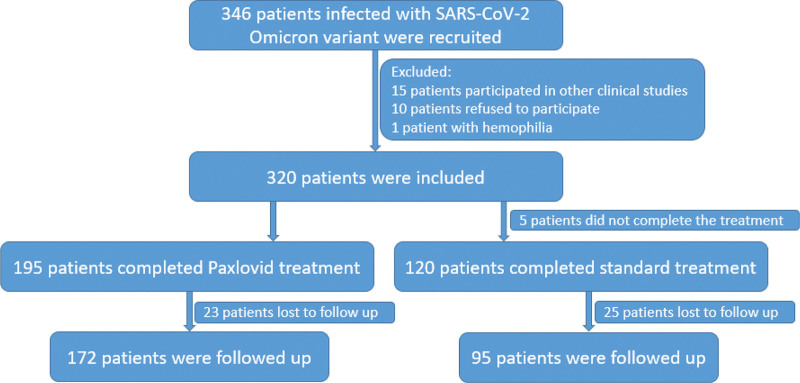
Follow chart.

A total of 346 patients infected with SARS-CoV-2 Omicron variant were recruited, with 15 patients participated in other clinical studies, 10 patients refused and 1 patient with hemophilia. Finally, a total of 320 patients were included, with mean age of 29.10 ± 7.34 years old. There were 200 patients receiving Paxlovid. During the treatment, 3 patients in the Paxlovid group refused to take the medication due to its taste, and 2 patients withdrew due to transfer to another hospital. A total of 315 patients completed the treatment (Fig. 1). Baseline characteristics included the sex, age, height, weight, body mass index, Ct values of positive nucleic acid N gene, Ct values of positive nucleic acid ORF gene, days from onset to admission and first clinical symptoms were comparable between patients with standard treatment and Paxlovid (all *P* > .05) (Table 1).

**Table 1:** Baseline characteristic of participants on admission.

Variables	Total	Paxlovid (n = 200)	Standard treatment (n = 120)	*P*
Baseline demographics				
Male, n (%)	192 (60%)	122 (61%)	70 (58%)	.637
Female, n (%)	182 (40%)	78 (39%)	50 (42%)	
Age, yr	29.10 ± 7.34	29.18 ± 7.33	28.96 ± 7.34	.787
Height (cm)	171.49 ± 8.15	172.02 ± 8.22	170.63 ± 7.99	.120
Weight (kg)	67.43 ± 11.83	68.24 ± 11.58	66.07 ± 12.18	.178
BMI (kg/m^2^)	22.77 ± 2.51	22.92 ± 2.58	22.52 ± 2.40	.219
CT at the baseline				
CT. N*	30.02 ± 2.91	29.81 ± 3.15	30.37 ± 2.42	.375
CT. ORF†	30.36 ± 2.20	30.40 ± 2.10	30.30 ± 2.37	.668
D from onset to admission‡	2.26 ± 1.51	2.38 ± 1.64	2.05 ± 1.24	.115
First symptoms, n (%)				
Fever	189 (59.06%)	124 (62.00%)	65 (54.17%)	.168
Cough	219 (68.44%)	139 (69.50%)	80 (66.67%)	.598
Expectoration	34 (10.63%)	18 (9.00%)	16 (13.33%)	.223
Sore throat	92 (28.75%)	60 (30.00%)	32 (26.67%)	.524
Fatigue	55 (17.19%)	33 (16.50%)	22 (18.33%)	.674
Headache	34 (10.63%)	18 (9.00%)	16 (13.33%)	.185
Stuffiness	35 (10.94%)	21 (10.50%)	14 (11.67%)	.746
Nasal discharge	29 (9.06%)	16 (8.00%)	13 (10.83%)	.393
Vomiting	17 (5.31%)	9 (4.50%)	8 (6.67%)	.403
Diarrhea	18 (5.63%)	9 (4.50%)	9 (7.50%)	.259
Limb pain/joint pain	29 (9.06%)	15 (7.50%)	14 (11.67%)	.209
Smell disorder	16 (5.00%)	11 (5.50%)	5 (4.17%)	.596

* Real-time PCR Ct value.

† Time from onset to enrolment in patients, including the time of initial symptoms or the first positive nucleic acid.

‡ According to WHO criteria.

BMI = body mass index, CT. N = Ct values of positive nucleic acid N gene, CT. ORF = Ct values of positive nucleic acid ORF gene, Paxlovid = Nirmatrelvir plus ritonavir.

**Figure 2.**
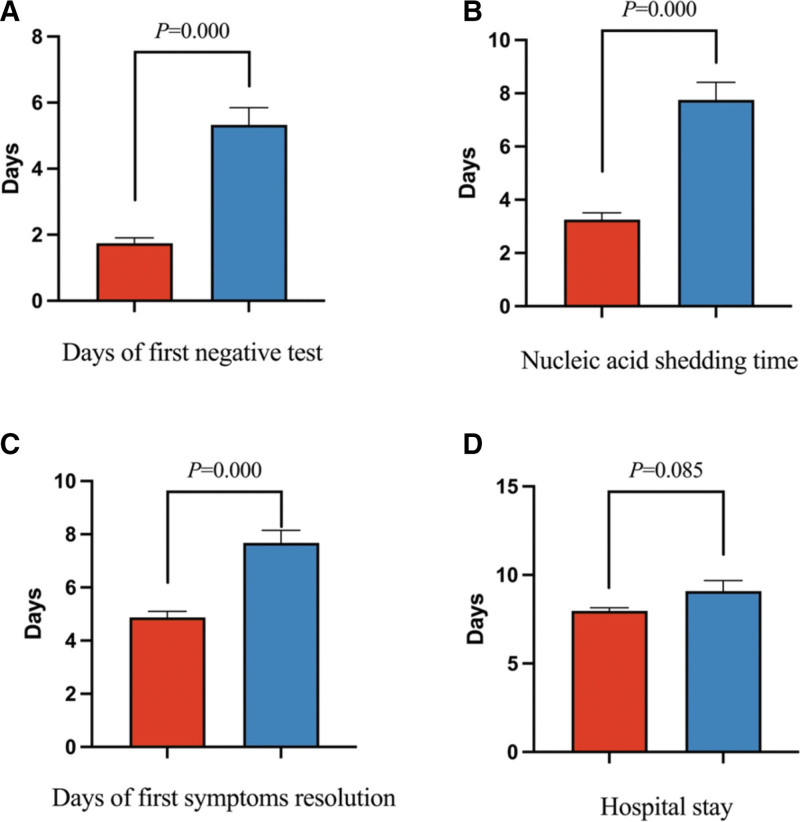
The major clinical outcomes of Paxlovid treatment in adult patients. Data are presented as means and 95% confidence intervals. (A) D until negative swab test. (B) Nucleic acid shedding time. (C) D of first symptoms resolution. (D) Hospital stay.

**Figure 3.**
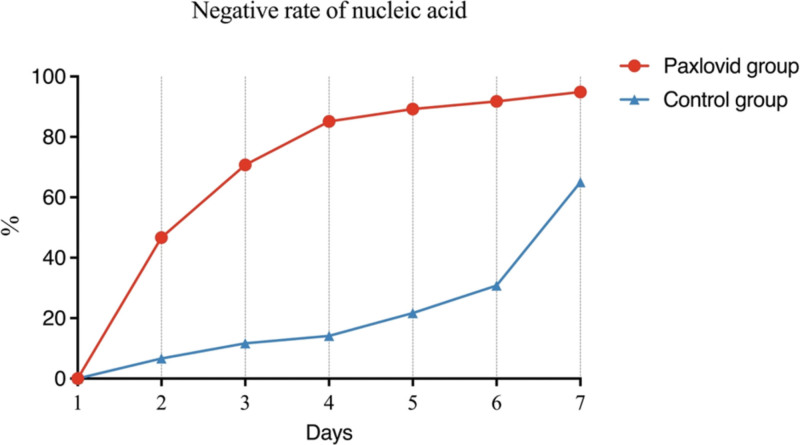
Line chart for the negative rate of nucleic acid.

Compared to patients with standard treatment, patients with Paxlovid had a significantly shorter nucleic acid shedding time (3.26 ± 1.80 vs 7.75 ± 3.68, *P* < .001), days until negative swab test (1.74 ± 1.15 vs 5.33 ± 2.91, *P* < .001), days of first symptoms resolution (4.86 ± 1.62 vs 7.45 ± 2.63, *P* < .001), as well as higher nucleic acid test negative rate within 3 days [138 (70.77%) vs 14 (11.67%), *P* < .001], 5 days [174 (89.23%) vs 26 (21.67%), *P* < .001], and 7 days [185 (94.87% vs 78 (65.00%), *P* < .001)]. However, there was no significant difference in hospital stay between patients with Paxlovid and standard treatment (7.97 ± 1.26 vs 9.08 ± 3.33 days, *P* = .085) (Table 2, Figs. 2 and 3).

**Table 2:** Outcomes for infected patients with Omicron variants.

	Paxlovid (n = 195)	Standard treatment (n = 120)	*P*
D until negative swab test (d)	1.74 ± 1.15	5.33 ± 2.91	<.001
Nucleic acid shedding time (d)	3.26 ± 1.80	7.75 ± 3.68	<.001
D of first symptoms resolution (d)	4.86 ± 1.62	7.45 ± 2.63	<.001
Hospital stay (d)	7.97 ± 1.26	9.08 ± 3.33	.085
Negative rate within 3 d, n (%)	138 (70.77%)	14 (11.67%)	<.001
Negative rate within 5 d, n (%)	174 (89.23%)	26 (21.67%)	<.001
Negative rate within 7 d, n (%)	185 (94.87%)	78 (65.00%)	<.001

Paxlovid: Nirmatrelvir plus ritonavir.

During the follow-up period, a total of 62 patients (24.72%) had post-COVID-19 condition within 3 months after discharge. There was significant difference in post-COVID-19 condition [32 (18.60%) vs 30 (31.57%), *P* = .016] and no significant difference in duration of post-COVID-19 condition (43.00 ± 26.00 vs 49.00 ± 26.34 days, *P* = .354) between Paxlovid and standard treatment groups. There was no significant difference in cough, fatigue, palpitation, hypomnesia, smell disorder, hair loss, taste disorder, headache, and insomnia between the 2 groups (all *P* > .05) (Table 3). Moreover, multivariable logistic regression analysis showed that, after adjusted for sex, age, height, weight, CT.N, CT.ORF, days from onset to admission, patients received Paxlovid [odds ratio = 0.531 (95%CI: 0.290–0.974), *P* = .041] would have a significant negative impact on the occurrence of post-COVID-19 condition. Multivariable Cox proportional hazards regression model analysis showed that, after adjusted for confounders above, patients received Paxlovid or standard treatment [hazards ratio = 1.300 (95% CI, 0.777–2.175), *P* = .317] had no significantly impact on the duration of post-COVID-19 symptoms (Table 4).

**Table 3:** Outcomes of follow-up study.

	Total (N = 267)	Paxlovid (N = 172)	Standard treatment (N = 95)	*P*
Baseline demographic				
Male, n (%)	160 (59.93%)	105 (61.05%)	55 (57.89%)	.615
Female, n (%)	107 (40.07%)	67 (39.95%)	40 (42.11%)	
Age (yr)	29.28 ± 7.60	29.37 ± 7.50	29.12 ± 7.85	.669
Height (cm)	171.59 ± 8.08	171.96 ± 8.18	170.93 ± 7.90	.286
Weight (kg)	67.52 ± 11.75	68.20 ± 11.51	66.28 ± 12.14	.279
BMI (kg/m^2^)	22.79 ± 2.55	22.93 ± 2.62	22.53 ± 2.41	.268
Post COVID-19 condition incidence rate				.016
No, n (%)	205 (75.28%)	140 (81.40%)	65 (68.43%)	
Yes, n (%)	62 (24.72%)	32 (18.60%)	30 (31.57%)	
Duration of post-COVID-19 condition (d)	45.73 ± 26.13	43.00 ± 26.00	49.00 ± 26.34	.354
Post-COVID-19 condition				
Cough, n (%)	33 (53.23%)	17 (53.13%)	16 (53.44%)	.885
Fatigue, n (%)	25 (40.32%)	13 (40.63%)	12 (40.00%)	.632
Palpitation, n (%)	13 (20.97%)	7 (21.88%)	6 (20.00%)	.856
Hypomnesia, n (%)	4 (6.45%)	3 (9.38%)	1 (3.33%)	.652
Smell disorder, n (%)	3 (4.84%)	2 (6.25%)	1 (3.33%)	.525
Hair loss, n (%)	3 (4.84%)	2 (6.25%)	1 (3.33%)	.525
Taste disorder, n (%)	2 (3.23%)	1 (3.13%)	1 (3.33%)	.963
Headache, n (%)	2 (3.23%)	1 (3.13%)	1 (3.33%)	.963
Insomnia, n (%)	2 (3.23%)	1 (3.13%)	1 (3.33%)	.963

BMI = body mass index, COVID-19 = coronavirus disease 2019, Paxlovid = Nirmatrelvir plus ritonavir.

**Table 4:** Multivariable analysis.

	Multivariable logistic regression analysis for post-COVID-19 condition		Multivariable Cox proportional hazards regression model analysis for duration of symptoms of COVID-19	
	OR (95%CI)	*P*	HR (95%CI)	*P*
Standard treatment	Ref	–	Ref	-
Paxlovid	0.531 (95%CI: 0.290–0.974)	.041	1.300 (95% CI, 0.777–2.175)	.317

CI = confidence interval, COVID-19 = coronavirus disease 2019, HR = hazards ratio, OR = odds ratio, Paxlovid = Nirmatrelvir plus ritonavir.

Adjusted model: sex, age, height, weight, CT.N, CT.ORF, d from onset to admission.

**Figure 4.**
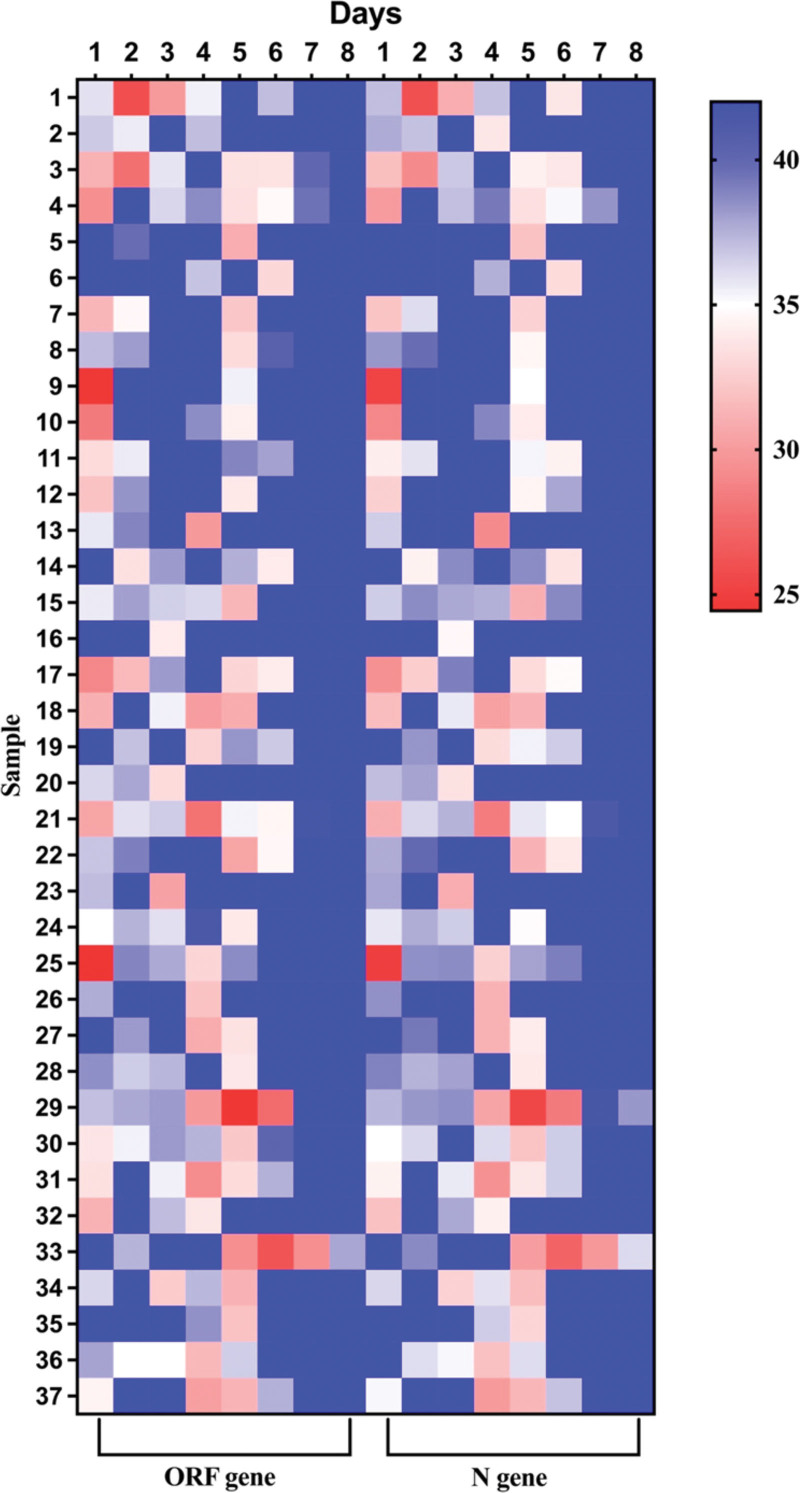
Heat map of the Ct values of ORF and N genes in Paxlovid group patients’ nucleic acid testing within 8 d.

After analyzing the nucleic acid results of patients with Paxlovid, some patients had 2 consecutive negative nucleic acid results or N gene and ORF gene Ct values ≥ 35 (with at least 24 hours between samplings), but later they tested positive again in the subsequent nucleic acid test (Fig. 4). Through the analysis of nucleic acid results of 195 patients in the Paxlovid group, the results showed that 37 (18.97%) patients had the above phenomenon, the median time for the 37 patients to test positive again was 5 days (3–8 days). Among them, 9 patients had 2 consecutive positive nucleic acid results and 2 patients had 3 consecutive positive nucleic acid results. The average Ct value of N gene for the positive nucleic acid results in the 37 patients was 32.75 ± 2.12, and the average Ct value of ORF gene was 32.76 ± 2.55. However, during the process of nucleic acid re-positivity, none of the 37 patients showed aggravation of major clinical symptoms.

No serious adverse event or death occurred in the 2 groups during the treatment progress. In the Paxlovid group, 12 patients (6%) experienced a bitter taste in their mouth, including 3 patients (1.5%) who refused to continue taking the medication and dropped out of the study. Additionally, 3 patients (1.5%) experienced gastrointestinal discomfort, and 2 patients (1%) experienced headaches. All these symptoms disappeared after the medication course was completed.

## 4. Discussion

This study showed that Paxlovid significantly reduced nucleic acid shedding time, days until negative swab test, and days of first symptoms resolution, as well as improved nucleic acid test negative rate and post-COVID-19 condition of Chinese adult patients who infected with SARS-CoV-2 Omicron variant compared with standard treatment, suggesting the good clinical efficacy of Paxlovid in treating patients infected with Omicron variant.

In this study, some patients who had Paxlovid showed a phenomenon of short-term re-detection of nucleic acid after turning negative. The median time of reappearance of positive result was the same as the treatment course of Paxlovid. Paxlovid mainly works by inhibiting the 3C-like protease of the virus,^[11]^ and it is possible that the drug ability to suppress the virus may weaken after the treatment course, allowing the virus to replicate again. It is worth noting that there are currently reports on the rebound of viral load after Paxlovid treatment, mainly retrospective study or clinical case reports,^[19]^ and there is currently no systematic analysis of the reasons for nucleic acid re-detection after drug treatment. Widespread and indiscriminate use of Paxlovid is controversial, since it may pose risk of developing drug resistance similar to antibiotic resistance.^[10]^ Prescription of Paxlovid should be limited to prevent severe COVID-19 in high risk patients, and other cheaper and more effective approaches to reduce infectivity of COVID-19 are available.^[20,21]^ Regarding other antiviral therapies, the effectiveness of Molnupiravir was equivalent to Paxlovid,^[10]^ and VV116 was non-inferior to Paxlovid.^[16]^

Data from 267 successfully followed-up cases found that the proportion of post-COVID-19 condition in Omicron variant-infected individuals is significantly lower than the previous observational study results on post-COVID-19 condition.^[22,23]^ This might be related with the different strains of the COVID-19 virus, as evidence has shown that the virulence and pathogenicity of the Omicron variant are significantly lower than those of the original strain and the Delta variant.^[24]^ Therefore, the proportion of post-COVID-19 condition in patients after negative nucleic acid test is lower than previous reports. Secondly, the participants included in this study were all young patients without underlying chronic diseases. Previous study on post-COVID-19 condition revealed a positive correlation between patients with underlying diseases and the occurrence of post-COVID-19 condition,^[25]^ which might explain for the lower proportion of post-COVID-19 condition in this study. The incidence of post-COVID-19 condition in the Paxlovid group was significantly lower than that in the standard treatment group, indicating that Paxlovid might reduce the incidence of post-COVID-19 condition in Omicron variant-infected individuals. Post-COVID-19 condition acts as the inflammatory response of the body when the virus invades,^[26]^ and Paxlovid can quickly block the replication of the SARS-CoV-2, reduce the viral load, alleviate the transitional inflammatory response, thus effectively improve the clinical symptoms. Analysis showed that the occurrence of post-COVID-19 condition was related to the symptoms of patients during the acute phase of infection,^[27]^ and this study found that Paxlovid could quickly alleviate symptoms such as fever, sore throat, and cough in infected individuals, which may be one of the reasons for reducing the incidence of post-COVID-19 condition. However, for patients who had already experienced post-COVID-19 condition, there was no difference in the duration of symptoms between the 2 groups, indicating that Paxlovid could not shorten the duration of symptoms for patients who already had post-COVID-19 condition, and therefore could not prove its therapeutic effect on post-COVID-19 condition.

The main clinical symptoms of 320 patients infected with Omicron variant in this study were fever, cough, fatigue, and limb pain, which were also observed in studies on the previous variants.^[28]^ However, among the patients included in this study, expectoration was lower than previous SARS-CoV-2 variants, while sore throat was more common. Omicron was reported to replicate poorly in Calu-3 cell lines expressing high levels of transmembrane serine protease 2 (TMPRSS2), while Delta variant replicated well.^[29]^ TMPRSS2 mediated the activation of spike protein to induce ACE2-mediated endocytosis, and then initiated fusion pore formation.^[30]^ TMPRSS2 and ACE2 were co-expressed in type II lung cells, while Delta variant grew faster and replicated better in the lungs and throat of humans.^[31]^ Moreover, during the study, no serious adverse events or deaths occurred, indicating its good safety. The average length of hospital stay was not significantly different between the 2 groups. The main reason for this was to prevent the phenomenon of nucleic acid re-positivity in patients taking Paxlovid.^[32]^ Therefore, the hospitalization time for patients in the Paxlovid group was artificially extended. All enrolled patients underwent nucleic acid testing at least 8 times and could only be discharged when the results of the last 2 nucleic acid tests met the discharge criteria. It is worth noting that this decision was fully communicated with the participants and obtained their informed consent.

There were also several limitations. First, the study was not randomized. Second, this study was a single-center study with small sample size, especially in the standard treatment group. Third, the participants were young adults, so the results could only be interpreted in this population. Fourth, due to the conditions of designated hospitals, the efficacy of drugs could only be evaluated based on changes in nucleic acid test results and clinical symptoms, lacking relevant laboratory indicators.

In conclusion, compared to standard treatment, Paxlovid effectively reduced the nucleic acid shedding time, speeded up the recovery of clinical symptoms, and improved post-COVID-19 condition of Chinese adult patients who infected with SARS-CoV-2 Omicron variant. Further randomized clinical trial with large sample size is warranted to further validate the results.

## Author contributions

**Data curation:** Jianchao Xu, Jinzhong Song, Ziyu Xie, Jie Yang, Di Wu.

**Formal analysis:** Ziyu Xie, Jie Yang, Di Wu.

**Investigation:** Fengshuang Liu, Yinuo Zhao, Hongmin Zang.

**Methodology:** Yubin Zhao.

**Writing – original draft:** Jianchao Xu, Jinzhong Song, Yubin Zhao.
